# Quantification of locomotor activity in larval zebrafish: considerations for the design of high-throughput behavioral studies

**DOI:** 10.3389/fncir.2013.00109

**Published:** 2013-06-10

**Authors:** Justin J. Ingebretson, Mark A. Masino

**Affiliations:** Department of Neuroscience, University of MinnesotaMinneapolis, MN, USA

**Keywords:** locomotor activity, high-throughput screening assays, larval zebrafish, measurement, model organism, development

## Abstract

High-throughput behavioral studies using larval zebrafish often assess locomotor activity to determine the effects of experimental perturbations. However, the results reported by different groups are difficult to compare because there is not a standardized experimental paradigm or measure of locomotor activity. To address this, we investigated the effects that several factors, including the stage of larval development and the physical dimensions (depth and diameter) of the behavioral arena, have on the locomotor activity produced by larval zebrafish. We provide evidence for differences in locomotor activity between larvae at different stages and when recorded in wells of different depths, but not in wells of different diameters. We also show that the variability for most properties of locomotor activity is less for older than younger larvae, which is consistent with previous reports. Finally, we show that conflicting interpretations of activity level can occur when activity is assessed with a single measure of locomotor activity. Thus, we conclude that although a combination of factors should be considered when designing behavioral experiments, the use of older larvae in deep wells will reduce the variability of locomotor activity, and that multiple properties of locomotor activity should be measured to determine activity level.

## Introduction

The use of larval zebrafish as a model organism in high-throughput behavioral screens is rapidly expanding due, at least in part, to the development and availability of commercial and academic analytical platforms designed to assess locomotor activity in larval zebrafish (Lessman, 2002; Anichtchik et al., 2004; Lockwood et al., 2004; Zon and Peterson, 2005; Giacomini et al., 2006; Prober et al., 2006; Burgess and Granato, 2007; Cahill, 2007; Parng et al., 2007; Winter et al., 2008; Creton, 2009; MacPhail et al., 2009; Sallinen et al., 2009a,b; Irons et al., 2010; Sylvain et al., 2010; Ali et al., 2011; Cario et al., 2011; Chen et al., 2011; Farrell et al., 2011; Padilla et al., 2011; Pelkowski et al., 2011; Cowden et al., 2012; Irons et al., 2013). Several characteristics of zebrafish embryos/larvae make them amenable to high-throughput behavioral screens. A high degree of conservation of the nervous system is present between zebrafish and mammals, so comparative studies are possible (Postlethwait et al., 2000; Higashijima et al., 2004; Kimura et al., 2006; Panula et al., 2010; Eklöf-Ljunggren et al., 2012). The small size of zebrafish larvae (~4 mm in length) and large number of embryos produced from a single mating permits high-throughput testing of large numbers of animals and experimental conditions simultaneously. Finally, zebrafish larvae are useful for chemical and pharmacological/toxicological screens, as they are permeable to small molecules (Kokel et al., 2010; Rihel et al., 2010; Peterson and Fishman, 2011; Tan and Zon, 2011).

There has not been, however, a systematic characterization of how experimental conditions affect the locomotor activity in larval zebrafish in the context of high-throughput testing. Evaluation of the current methods used for high-throughput studies has led us to propose that several factors, which vary between research groups, could affect locomotor activity or the conclusions drawn from such studies. First, large sample-sizes are necessary due to the recognized high degree of intra-larval variability in the locomotor activity (Colwill and Creton, 2011; Farrell et al., 2011; Lambert et al., 2012). Second, assessing activity by measuring and reporting a single property of locomotor activity (i.e., swim speed or total distance) may lead to an incomplete understanding of activity level. For example, different groups of larvae can produce similar total distances traveled per time by generating different swim durations and/or swim speeds. This strongly suggests that multiple properties of locomotor activity must be examined and integrated to fully depict activity levels. Third, skeletal deformities, such as tail kinks are often observed when zebrafish embryos/larvae are reared in multiwell plates (Selderslaghs et al., 2009; Padilla et al., 2011). Such deformities can affect how larvae locomote and, if these larvae are eliminated from the study, can reduce throughput. Finally, the ratio of larval body length to behavioral well diameter (length-to-diameter) may alter the properties of locomotor activity. For example, when the ratio nears one (length and diameter are nearly equivalent, as in 96-well plates), space constraints may affect the speed and duration of locomotion, but not when the ratio is less than one (length is smaller than well diameter, as in 6, 12, 24, or 48 well plates). Recent reports showed that the locomotor activity was different for larvae in 96 well and 24-well plates (Farrell et al., 2011; Padilla et al., 2011), but did not examine a range of well dimensions to determine the effect of diameter on locomotor activity.

To date, these factors have not been fully taken into consideration for the experimental design or data interpretation of locomotor activity. Thus, the goal of this study was to determine the effects of these factors in an attempt to identify and standardize the parameters necessary to assess locomotor activity.

## Materials and methods

### Animals

All experiments were performed on zebrafish (*Danio rerio*) larvae at 4 and 7 days post-fertilization (dpf). Wild-type larvae were obtained from a laboratory stock (Segrest Farms; Gibsonton, FL) of adults at the University of Minnesota. Embryos and larvae were maintained in petri dishes (100 mm dia) filled with embryo water (60 μg/mL Instant Ocean salt mix; Cincinnati, OH) in a 28.5°C incubator under a 14/10 h light/dark cycle until the start of behavioral recordings at 4 or 7 dpf. The density was not greater than 60 embryos or larvae per dish. Larvae hatched spontaneously between 2 and 3 dpf and were not fed until 8 dpf; one day after the final video recording at 7 dpf. Only larvae with inflated air bladders at 4 dpf were selected for these studies. Larvae were kept at room temperature during recording sessions. All procedures were approved by the Institutional Animal Care and Use Committee at the University of Minnesota and were in accordance with National Institutes of Health guidelines.

### Video acquisition

To acquire video recordings of free-swimming behavior and to eliminate group interactions, larvae were placed individually in wells (see below) containing embryo media on custom-built plates. The plates were composed of either acetal resin (L × W × D: 165 × 102 × 1.5 mm; Delrin, DuPont) or acrylic plastic (L × W × D: 165 × 102 × 5.5 mm; polymethyl methacrylate, PlexiGlas); both materials were black in color. The plates were secured to a piece of glass (L × W × D: 178 × 114 × 2 mm) with dental cement (polyvinylsiloxane impression material Type I, medium viscosity #3604-14952, Kerr Manufacturing Company). Each plate was comprised of six arenas (“wells”) with different combinations of depth [1.5 mm (shallow) or 5.5 mm (deep)] and diameter [10 mm (small), 20 mm (medium), or 30 mm (large)]. The wells were made with a computer-controlled laser-cutter, which left the inner surface of the well with a matte-like finish. All six wells on an individual plate were composed of a single depth and a single diameter. The following volumes of embryo water were used to fill the wells: 150 μL (1.5 mm depth × 10 mm dia.), 600 μL (1.5 mm depth × 20 mm dia.), and 1.8 mL (1.5 mm depth × 30 mm dia.); 500 μL (5.5 mm depth × 10 mm dia.), 2.0 mL (5.5 mm depth × 20 mm dia.), and 4.1 mL (5.5 mm depth × 30 mm dia.). Due to the convex meniscus formed, the depth of the shallow wells was ~2 mm and the deep wells was ~6 mm. Depths were chosen to bound the vertical range over which the larvae could access; either to restrict locomotion to two-dimensions (*x* and *y*; shallow) or to allow access to the vertical dimension (*z*; deep) of the well, while diameters were chosen based on commercially available well plates typically used for locomotor assessment (48 well: 10.5 mm; 12 well: 22.1 mm; 6 well: 34.8 mm; Creton, 2009; Sallinen et al., 2009a,b; Selderslaghs et al., 2009; Cario et al., 2011; Chen et al., 2011; Colwill and Creton, 2011; Farrell et al., 2011; Padilla et al., 2011). We did not assess activity in 96 well plates (6.8 mm well diameter), in which the larval body length to behavioral well diameter ratio was near one, because others have previously reported that space constraints affect locomotor activity (Selderslaghs et al., 2009; Farrell et al., 2011; Padilla et al., 2011). The plates were positioned atop a transmitted LED light stage (Metaphase Technologies) and a light meter (Extech Instruments) was used to measure the light intensity at the level of the arena, which was 4 Klx to maximize contrast and facilitate tracking of dark targets on a light background. For all experiments, testing occurred between 9 am and 4 pm using a randomized trial design to eliminate systematic effects due to time of day. The larvae acclimated to the recording arena for 10 min before the start of video acquisition. Subsequently, video of spontaneous free-swimming was recorded for 10 min at 60 frames/s using a digital CMOS camera (Firefly MV; Point Grey Research) with an attached 12 mm lens (Navitar). The camera was mounted to a copystand and videos were acquired and saved without compression, via Fview (Straw and Dickinson, 2009).

### Tracking and analysis

The videos generated by Fview were analyzed to obtain independent trajectories of each target within the arena as previously described (Lambert et al., 2012). We used the open-source Fix Errors Matlab Toolbox (FEMT), provided by the creators of Ctrax (Branson et al., 2009), to identify and fix tracking errors, such as loss of target or false target recognition. The total number of errors per 10-min video was 0.06 ± 0.03 (*n* = 288 videos) and all errors were corrected via the FEMT. Scripts from the open-source Behavioral Microarray MATLAB Toolbox (Branson et al., 2009) were used to compute a suite of behavioral parameters for each of the individual targets from the fixed Ctrax trajectories. The speed of the center of rotation was extracted for each target to define and detect swimming event onsets and offsets, as described previously (Lambert et al., 2012), with minor modifications; we used a 2.0 mm/s speed threshold filter and a minimum 10 frame (166.6 ms) inter-event interval (end-to-start). Episodes of swimming were defined as the activity between event onsets and offsets. Identical filters were applied for all videos across all developmental stages and well dimensions. Next, the speed of the center of mass was extracted for each target to determine the instantaneous velocities during swimming episodes. Larvae were considered “motile” when at least one swimming episode was identified during the 10 min recording and “non-motile” when swimming episodes were absent.

The properties of locomotor activity that we measured were: (1) Episode Frequency [number of episodes per sec (Hz)], (2) Episode Duration [duration of time between events onset and offset (ms)], (3) Swim Speed [mean instantaneous velocity per episode (mm/s)], (4) Active Swim Time [sum of the number of frames between events onset and offset divided by the frame rate (s)], and (5) Total Distance [summed instantaneous speeds during swimming episodes divided by frame rate (cm)] and measured for each larva. All measures were then averaged across larvae within each condition and reported as population means ± SD.

### Ethanol treatment

Ethanol (200 proof, undenatured, Decon Laboratories) was prepared at a 1% concentration (v/v) in embryo media. This ethanol concentration was selected on the basis of pilot studies (data not shown) and previous reports (Lockwood et al., 2004; Gerlai et al., 2009; MacPhail et al., 2009; Irons et al., 2010; Chen et al., 2011; Cowden et al., 2012). The effects of acute ethanol exposure on locomotor activity in larval zebrafish were examined using the experimental paradigm described above (see Video Acquisition). However, we restricted the factors in this analysis to include only 7 dpf larvae in deep wells; all well diameters were used. As described above, the larvae acclimated to the behavioral arena and then a 10 min video of spontaneous free-swimming was acquired. The larvae were then exposed to ethanol (1%) for 30 min followed by a second 10 min video of spontaneous free-swimming; larvae remained in ethanol during video acquisition. Only larvae that were motile in both the control and treatment conditions were used for this analysis.

### Statistical analysis

Statistical analyses were performed with SigmaPlot 12.0 (Systat Software). Motile larvae were analyzed using Fisher's *z*-coefficient to test for significant differences between proportions. The main effects of stage, depth and diameter on locomotor activity were tested for significance using a Three-Way ANOVA. For the ethanol study, a repeated measures ANOVA was used. *Post-hoc* tests were performed using the Holm–Sidak correction for multiple comparisons. Significance was established using an α criterion of *p* = 0.05. To estimate the variability of the measures of locomotor activity the group coefficients of variation (CoVs) were calculated as the group standard deviation (SD) divided by the group mean.

## Results

### Locomotor activity differs between larvae at distinct developmental stages

**Figure 1:**
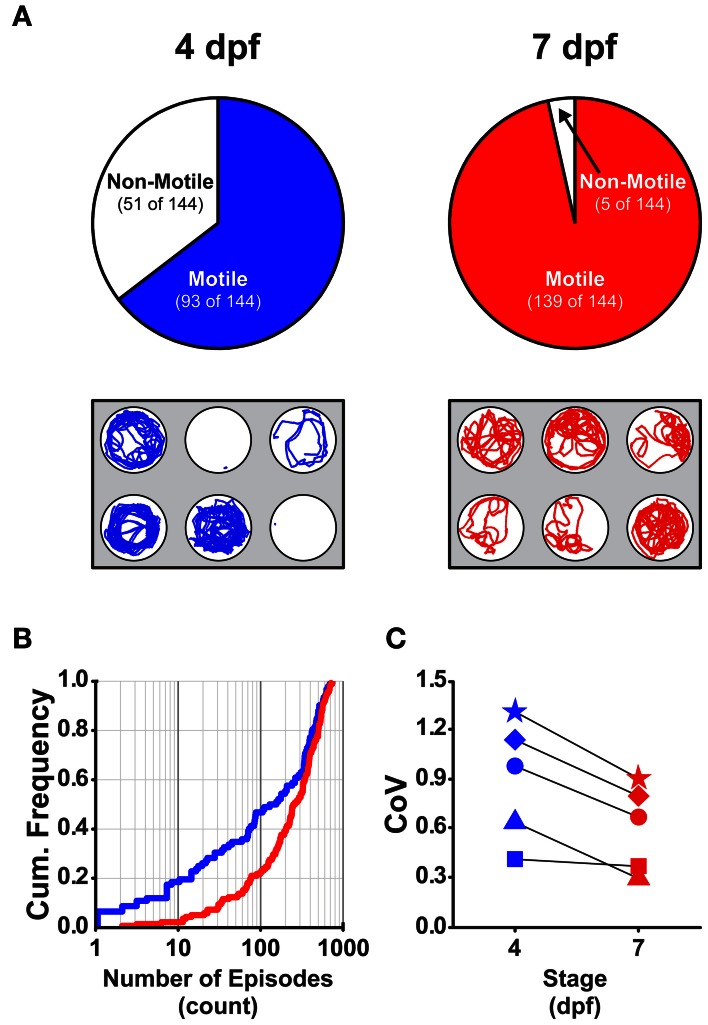
**The proportion of motile larvae and the variability among the locomotor properties are different between 4 and 7 dpf larvae. (A)** *Top Row*, Pie charts depicting the proportions of motile and non-motile larvae at 4 dpf and 7 dpf (blue and red, respectively). *Bottom Row*, Representative trajectory plots for larvae in 6-well behavioral arena plates (30 mm well diameter shown); single larva per well. **(B)** Cumulative frequency plots for the number of swim episodes produced by 4 dpf and 7 dpf larvae (blue and red, respectively). **(C)** Plots of the group coefficients of variation (CoV) for all measured locomotor properties at 4 dpf (*n* = 93) and 7 dpf (*n* = 139) (blue and red, respectively). Symbols are: circle, episode frequency; square, episode duration; triangle, swim speed; diamond, active swim time; star, total distance.

Previous studies showed that larvae at distinct developmental stages produce different locomotor activities (Saint-Amant and Drapeau, 1998, 2000; Budick and O'Malley, 2000; Drapeau et al., 2002; Colwill and Creton, 2011; Lambert et al., 2012; Tong and McDearmid, 2012). We examined locomotor activity produced by larvae at 4 and 7 dpf because larvae at these stages of development produce beat-and-glide swimming (Buss and Drapeau, 2001; Drapeau et al., 2002). Larvae at earlier and later developmental stages were excluded from this study because they produce either burst locomotion at 3 dpf (Saint-Amant and Drapeau, 1998; Budick and O'Malley, 2000; Buss and Drapeau, 2001; Drapeau et al., 2002) or subtle changes to the locomotor activity between 5 and 8 dpf (Müller and van Leeuwen, 2004; Colwill and Creton, 2011; Farrell et al., 2011), respectively. As a first approximation of locomotor activity, we determined the proportions of 4 and 7 dpf larvae (*n* = 144 for both groups) that were motile (see Materials and Methods) across all well depths (deep and shallow) and diameters (small, medium, and large). A significantly higher proportion of larvae were motile at 7 dpf than at 4 dpf (97 and 65%, respectively; Fisher's *z*-coefficient = 6.64, *p* < 0.005; Figure 1A), which was consistent with previous results from our lab (Lambert et al., 2012). Next, we determined whether developmental stage affected the locomotor properties of larvae. Larvae at 7 dpf produced significantly greater episode frequency (Figure 1B), shorter episode duration, slower swim speed, less active swim time and less total distance than did 4 dpf larvae (Table 1). Note that these measures of the properties of locomotor activity are not independent from one another. Finally, to determine whether the variability measured in the locomotor properties was affected by the developmental stage of the larvae, we calculated the coefficient of variation (CoV) for each measure and compared them across developmental stages (4 and 7 dpf). The CoVs of all properties except episode duration were smaller for 7 dpf larvae than for 4 dpf larvae (Figure 1C). These results showed that developmental stage affected motility and the properties and the variability of locomotor activity.

**Table 1:** **Comparison of the properties of locomotor activity for larvae at 4 and 7 dpf**.

**Stage**	**Episode frequency (Hz)**	**Episode duration (ms)**	**Swim speed (mm/s)**	**Active swim time (s)**	**Total distance (cm)**
4 dpf	0.36 ± 0.36	196.5 ± 87.6	8.0 ± 5.2	44.5 ± 50.3	38.8 ± 50.1
7 dpf	0.48 ± 0.34	114.3 ± 46.7	6.7 ± 2.2	32.8 ± 26.5	21.9 ± 20.0
*F*	8.603	88.643	6.573	4.088	10.793
*p*	0.004	<0.001	0.011	0.044	0.001

Data expressed as group means ± SD. F, test statistic for Three-Way ANOVA; p, probability.

### Locomotor activity differs between larvae in wells of different depths

**Figure 2:**
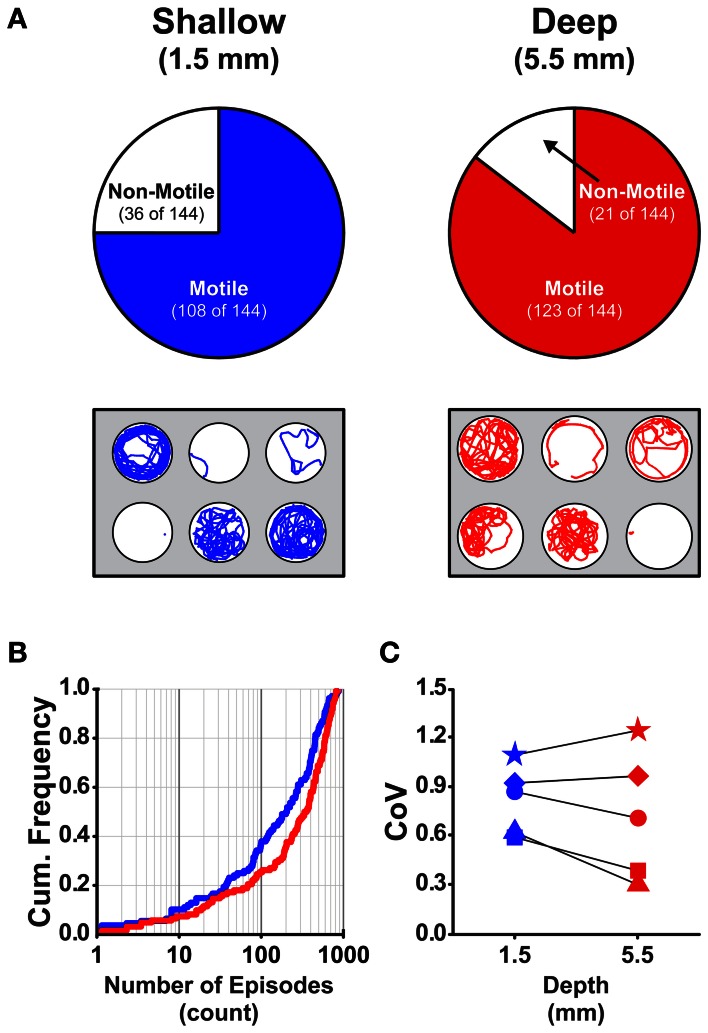
**The proportion of motile larvae and the variability among the locomotor properties are different for larvae in shallow and deep behavioral wells. (A)** *Top Row*, Pie charts depicting the proportions of motile and non-motile larvae in Shallow and Deep wells (blue and red, respectively). *Bottom Row*, Representative trajectory plots for larvae in 6-well behavioral arena plates (30 mm well diameter shown); single larva per well. **(B)** Cumulative frequency plots for the number of swim episodes produced by larvae in Shallow and Deep wells (blue and red, respectively). **(C)** Plots of the group coefficients of variation (CoV) for all measured locomotor properties in Shallow (*n* = 108) and Deep (*n* = 123) wells (blue and red, respectively). Symbols are: circle, episode frequency; square, episode duration; triangle, swim speed; diamond, active swim time; star, total distance.

Currently, there is a lack of information regarding how the dimensions (depth and diameter) of the behavioral arenas affect locomotor activity in larval zebrafish. We determined the proportions of larvae that were motile (see Materials and Methods) in shallow and deep wells (*n* = 144 for both groups) across all stages (4 and 7 dpf) and well diameters (small, medium, and large). A larger proportion of larvae were motile in deep wells than in shallow wells (85 and 75%, respectively; Fisher's *z*-coefficient = 2.13, *p* < 0.05; Figure 2A). Next, we determined whether well depth affected the locomotor properties of larvae. Larvae in deep wells produced significantly greater episode frequency (Figure 2B), active swim time and total distance than did larvae in shallow wells (Table 2). However, the episode durations and swim speeds were not significantly different (Table 2). Finally, to determine whether the variability measured in the locomotor properties was affected by well depth, we calculated the CoV for each measure and compared them across well depths. The CoVs of all properties except active swim time and total distance were smaller for larvae in deep wells than for larvae in shallow wells (Figure 2C). These results showed that well depth affected motility and the properties and variability of locomotor activity.

**Table 2:** **Comparison of the properties of locomotor activity for larvae in behavioral wells of different depths**.

**Depth**	**Episode frequency (Hz)**	**Episode duration (ms)**	**Swim speed (mm/s)**	**Active swim time (s)**	**Total distance (cm)**
Shallow	0.38 ± 0.32	147 ± 90.7	7.5 ± 4.9	28.9 ± 28.2	20.8 ± 22.7
Deep	0.50 ± 0.36	147.4 ± 63.6	6.8 ± 2.4	45.1 ± 43.9	35.6 ± 43.7
*F*	10.225	0.0214	2.421	13.762	13.112
*p*	0.002	0.884	0.121	<0.001	<0.001

Data expressed as group means ± SD. F, test statistic for Three-Way ANOVA; p, probability.

### Locomotor activity differs between larvae in wells of different diameters

**Figure 3:**
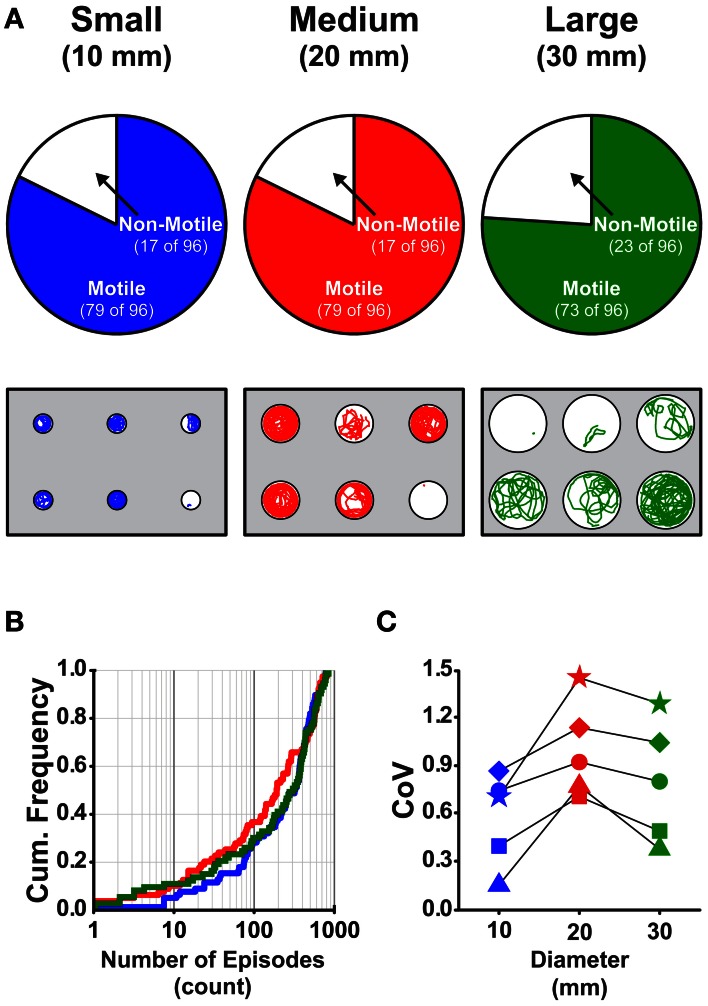
**The proportions of motile larvae are not different, but the variability among the locomotor properties is different for larvae in wells of different diameters. (A)** *Top Row*, Pie charts depicting the proportions of motile and non-motile larvae in Small, Medium, and Large diameter wells (blue, red, and green, respectively). *Bottom Row*, Representative trajectory plots for larvae in 6-well behavioral arena plates (10, 20, and 30 mm diameter wells shown); single larva per well. **(B)** Cumulative frequency plots for the number of swim episodes produced by larvae in Small, Medium, and Large diameter wells (blue, red, and green, respectively). **(C)** Plots of the group coefficients of variation (CoV) for all measured locomotor properties in Small (*n* = 79), Medium (*n* = 79), and Large (*n* = 73) diameter wells (blue, red, and green, respectively). Symbols are: circle, episode frequency; square, episode duration; triangle, swim speed; diamond, active swim time; star, total distance.

To test the effect of well diameter on locomotor activity in larval zebrafish, we determined the proportions of larvae that were motile (see Materials and Methods) in small, medium, and large diameter wells across all stages (4 and 7 dpf) and well depths (shallow and deep). The proportion of larvae that were motile did not vary with diameter; all pairwise comparisons produced Fisher's *z*-coefficients that were ≤ 1.02 and *p*-values that were ≥ 0.05 (Figure 3A). Next, we determined whether locomotor properties were different for larvae in behavioral wells of different diameters (small, medium, or large). We found that none of the measured locomotor properties were significantly different between groups (Table 3). Finally, to determine whether the variability measured in the locomotor properties was affected by well diameter, we calculated the CoV for each measure and compared them across diameters. The CoVs of all measured properties were smallest for larvae in small diameter wells and largest for larvae in medium diameter wells (Figure 3C). These results showed that well diameter did not affect larval motility or the measured properties of locomotor activity, yet the variability of some, but not all, measured properties of locomotor activity differed by well diameter.

**Table 3:** **Comparison of the properties of locomotor activity for larvae in behavioral wells of different diameters**.

**Diameter**	**Episode frequency (Hz)**	**Episode duration (ms)**	**Swim speed (mm/s)**	**Active swim time (s)**	**Total distance (cm)**
Small	0.45 ± 0.33	137.6 ± 47.5	7.5 ± 2.7	36.4 ± 31	28.5 ± 28
Medium	0.40 ± 0.36	153.2 ± 104.4	7.3 ± 5.5	34.4 ± 39.2	26.5 ± 39
Large	0.46 ± 0.36	150.9 ± 68.3	6.6 ± 2.2	41.9 ± 43.6	31.1 ± 40.5
*F*	0.662	2.793	1.868	0.780	0.386
*p*	0.517	0.063	0.157	0.460	0.680

Data expressed as group means ± SD. F, test statistic for Three-Way ANOVA; p, probability.

### Locomotor activity is affected by interactions between developmental stage and well depth

**Figure 4:**
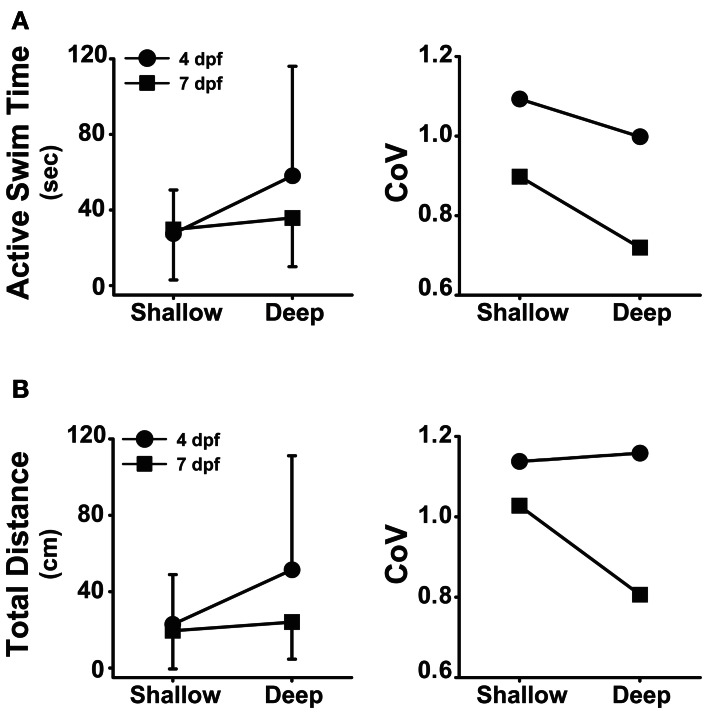
**Developmental stage and well depth interact to affect locomotor activity. (A)** *Left*, plot of the interaction between stage and depth for active swim time. *Right*, plot of the group coefficients of variation for each interaction condition (4 dpf × Shallow, *n* = 41; 4 dpf × Deep, *n* = 51; 7 dpf × Shallow, *n* = 67; 7 dpf × Deep, *n* = 71). **(B)** *Left*, plot of the interaction between stage and depth for total distance. *Right*, plot of the group coefficients of variation (CoV) for each interaction condition (4 dpf × Shallow, *n* = 41; 4 dpf × Deep, *n* = 51; 7 dpf × Shallow, *n* = 67; 7 dpf × Deep, *n* = 71). Measures of active swim time and total distance are expressed as mean ± SD.

In addition to the differences observed for developmental stage (Figure 1; Table 1) and well depth (Figure 2; Table 2), the analysis of variance revealed significant interactions between developmental stage and depth for both active swim time (*F* = 5.37, *p* = 0.02) and total distance (*F* = 6.31, *p* = 0.01). *Post-hoc* analyses revealed that larvae at 4 dpf spent more time swimming in deep wells than in shallow wells (*t* = 3.9, *p* < 0.001, *n* = 92; Figure 4A, left), while 7 dpf larvae spent similar amounts of time swimming in deep and shallow wells (*t* = 1.1, *p* = 0.27, *n* = 138; Figure 4A, left). In deep wells, 4 dpf larvae spent more time swimming than did 7 dpf larvae (*t* = 3.2, *p* = 0.002, *n* = 122; Figure 4A, left), while in shallow wells, 4 and 7 dpf larvae spent similar amounts of time swimming (*t* = 0.2, *p* = 0.84, *n* = 108; Figure 4A, left). The CoV was smallest for 7 dpf larvae in deep wells, and larger for all other combinations of stage and depth (Figure 4A, right). These results showed that, although 4 dpf larvae in deep wells spent the greatest amount of time swimming, 7 dpf larvae in deep wells produced the most reliable measure (lowest CoV) for active swim time.

*Post-hoc* analyses also revealed that larvae at 4 dpf traveled greater total distances in deep wells than in shallow wells (*t* = 3.9, *p* < 0.001, *n* = 92; Figure 4B, left), while 7 dpf larvae traveled similar total distances in deep and shallow wells (*t* = 0.9, *p* = 0.38, *n* = 138; Figure 4B, left). In deep wells, 4 dpf larvae traveled greater total distances than did 7 dpf larvae (*t* = 4.3, *p* < 0.001, *n* = 122; Figure 4B, left), while in shallow wells, 4 and 7 dpf larvae traveled similar total distances (*t* = 0.5, *p* < 0.001, *n* = 108; Figure 4B, left). The CoV was smallest for 7 dpf larvae in deep wells, and larger for all other combinations of stage and depth (Figure 4B, right). These results showed that 4 dpf larvae in deep wells traveled the greatest total distance and 7 dpf larvae in deep wells produced the least variation in total distance.

### Interpretation of activity level requires the assessment of multiple properties of locomotor activity

**Figure 5:**
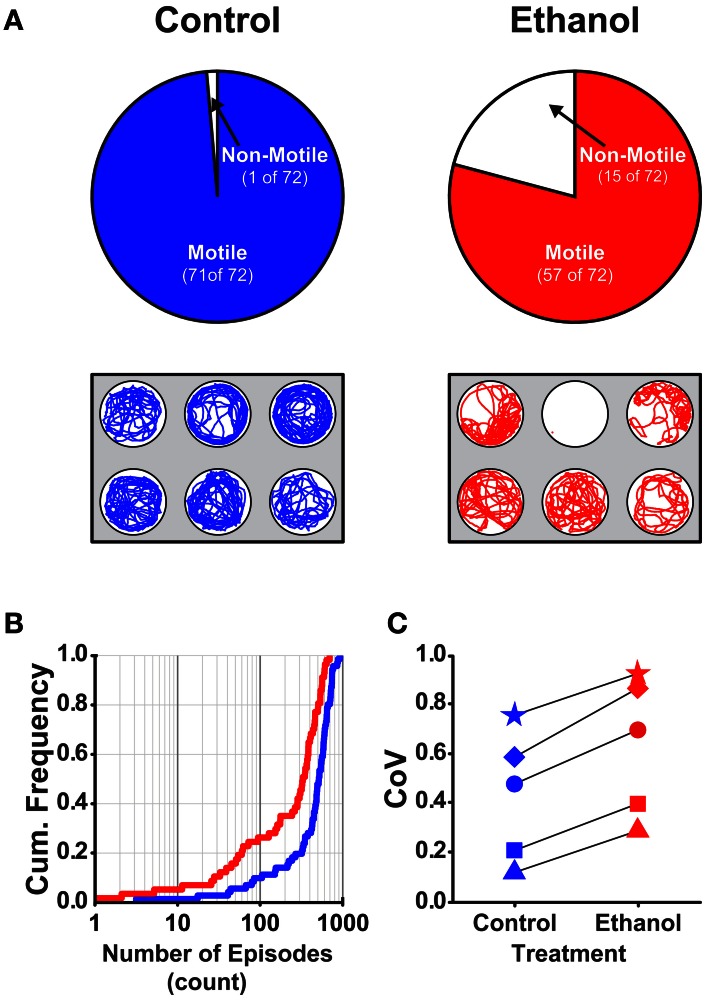
**The proportion of motile larvae and the variability among the locomotor properties are different for control and ethanol treated larvae (7 dpf in deep behavioral wells). (A)** *Top Row*, Pie charts depicting the proportions of motile and non-motile larvae in Control and Ethanol treated groups (blue and red, respectively). *Bottom Row*, Representative trajectory plots for larvae in 6-well behavioral arena plates (30 mm well diameter shown); single larva per well. **(B)** Cumulative frequency plots for the number of swim episodes produced by Control and Ethanol treated larvae (blue and red, respectively). **(C)** Plots of the group coefficients of variation (CoV) for all measured locomotor properties for Control (*n* = 71) and Ethanol (*n* = 57) treated groups (blue and red, respectively). Symbols are: circle, episode frequency; square, episode duration; triangle, swim speed; diamond, active swim time; star, total distance.

Previous studies that examined the effects of acute ethanol treatment on locomotor activity in zebrafish larvae report increased (hyper-) activity due to ethanol as assessed by comparing swim speeds or total distance traveled between control and ethanol treated larvae (Lockwood et al., 2004; MacPhail et al., 2009; Irons et al., 2010; Chen et al., 2011). This result was comparable to the effects on locomotor activity observed in mammals in response to acute ethanol exposure (Frye and Breese, 1981; Masur et al., 1988; Dudek et al., 1991; Phillips et al., 1991, 1992; Shen et al., 1995; Palmer et al., 2002; Addicott et al., 2007). To better understand how ethanol treatment affects locomotor activity in larval zebrafish, we examined all measured properties of locomotor activity (see Materials and Methods). We used larvae at 7 dpf in deep wells because this combination of factors produced the most reliable measures of the locomotor properties (Figures 1, 2, 4). Further, all well diameters were included because locomotor activity was not affected by diameter (Figure 3). A larger proportion of control larvae than ethanol treated larvae were motile (99 and 79%, respectively; Fisher's *z-coefficient* = 2.95, *p* < 0.01; Figure 5A). Significantly lower episode frequency (Figure 5B) and greater episode duration and faster swim speed were produced by ethanol treated larvae than by control larvae (Table 4). However, active swim time and total distance were not significantly different between groups (Table 4). Again, this suggests that the assessment of locomotor activity by any one of these properties alone was not sufficient to fully depict activity level. The CoVs for all properties were smaller for control larvae than for treated larvae (Figure 5C). These results indicated that ethanol treated larvae were hypoactive relative to control (non-treated) larvae when activity was assessed based on the episode frequency (Table 4). However, these results also indicated that ethanol treated larvae were hyperactive relative to control (non-treated) larvae when activity was assessed based on swim speed or episode duration (Table 4). Thus, depending on the locomotor property reported, differing interpretations of the activity level for treated larvae can be made.

**Table 4:** **Comparison of the properties of locomotor activity for larvae in control and ethanol treated groups**.

**Treatment**	**Episode frequency (Hz)**	**Episode duration (ms)**	**Swim speed (mm/s)**	**Active swimming (s)**	**Total distance (cm)**
Control	0.80 ± 0.38	115.4 ± 24.8	6.4 ± 1.3	57.2 ± 34.0	39.1 ± 29.9
Ethanol	0.50 ± 0.33	175.5 ± 69.6	7.6 ± 2.2	59.5 ± 51.7	47.8 ± 44.3
*F*	22.131	36.804	10.903	0.147	2.144
*p*	<0.001	<0.001	0.002	0.703	0.149

Data expressed as group means ± SD. F, test statistic for Three-Way ANOVA; p, probability.

## Discussion

Previous studies have shown that patterns of locomotor activity change during development (Saint-Amant and Drapeau, 1998, 2000; Budick and O'Malley, 2000; Drapeau et al., 2002; Colwill and Creton, 2011; Lambert et al., 2012; Tong and McDearmid, 2012). Here, we report that most 7 dpf larvae were motile, whereas significantly fewer larvae were motile at 4 dpf (Figure 1A). Further, all of the measured locomotor properties were significantly different between larvae at 4 and 7 dpf (Table 1), and were less variable for 7 dpf than for 4 dpf larvae (Figure 1C), which was consistent with a previous report (Farrell et al., 2011). We propose that the use of larvae at later developmental stages (e.g., 7 dpf) to assess locomotor activity will generate more reliable data as larvae at this stage produced more consistent locomotor activity.

Our data also showed that locomotor activity was sensitive to differences in well depth; most larvae were motile in deep wells, whereas significantly fewer larvae were motile in shallow wells (Figure 2A). In addition, significant differences were found for many of the measured locomotor properties produced between larvae in shallow and deep wells (Table 2). Finally, most of the measured locomotor properties were less variable for larvae in deep wells than in shallow wells (Figure 2C). Thus, we suggest the use of deep wells to assess locomotor activity because larvae in deep wells produced more reliable and consistent locomotor activity (e.g., 5.5 mm well depth reported here).

Locomotor activity was not sensitive, however, to differences in well diameter; ~80% of all larvae were motile in wells of all diameters examined (Figure 3A), and there were no significant differences for any the measured locomotor properties produced between larvae in wells of different diameters (Table 3). Most of the locomotor properties were less variable for larvae in small diameter wells than for larvae in either medium or large wells (Figure 3C). It was surprising to us that well diameter did not have a significant effect on locomotor activity. However, this result does provide a benefit for experimental design; specifically, for a given field of view set by the camera and lens combination, one can maximize the high-throughput capacity simply by decreasing the diameter of the wells and, therefore, increasing the number of wells per behavioral plate. There is a limit, however, to the reduction of the well diameter. When the length-to-diameter ratio of the embryonic/larval zebrafish to the behavioral well is near one, then the speed and duration of locomotor activity will be affected (Farrell et al., 2011; Padilla et al., 2011), and if the fish are reared in small diameter wells, there is potential for structural and morphological defects to arise, such as tail links (Selderslaghs et al., 2009; Padilla et al., 2011) that will also adversely affect the properties of locomotor activity.

### Considerations for the quantitative assessment of locomotor activity

Prior to this study it was shown that larval developmental stage affected locomotor activity, however, the effects of the dimensions of the behavioral arena were less well understood. We hypothesized that locomotor activity would be strongly influenced by several interacting factors, including developmental stage and the dimensions (depth and diameter) of the behavioral arena. Here, we showed that stage and depth not only affected locomotor activity (Figures 1, 2, respectively), but stage and depth also interacted to affect active swim time and total distance (Figure 4). In addition, 7 dpf larvae in deep wells produced the most consistent (lowest CoV) measures of active swim time and total distance (Figure 4). Together, these results suggest the use of older larvae in deep wells to assess locomotor activity.

For most current experimental paradigms, a single quantitative measure, such as swim speed or total distance, is used to assess locomotor activity in larval zebrafish (Vogl et al., 1999; Lockwood et al., 2004; Giacomini et al., 2006; MacPhail et al., 2009; Sallinen et al., 2009a; Selderslaghs et al., 2009; Padilla et al., 2011; Cowden et al., 2012; Irons et al., 2013). Although more recent studies have included measures of multiple parameters of locomotor activity (Anichtchik et al., 2004; Prober et al., 2006; Burgess and Granato, 2007; Sallinen et al., 2009b; Chou et al., 2010; Chen et al., 2011; Colwill and Creton, 2011; Sundvik et al., 2011), the critical step of integrating these parameters necessary to more fully assess and depict locomotor activity is missing. Our data showed that the reliance on a single measure of locomotor activity was not sufficient to assess global activity levels, but that a more thorough examination of multiple properties of locomotor activity may be necessary (Tables 1–3). For example, our results indicate that 7 dpf larvae are hyperactive relative to 4 dpf larvae when activity is assessed based on the episode frequency (Table 1). However, our results also indicate that 7 dpf larvae are hypoactive relative to 4 dpf larvae when activity is assessed based on the total distance (Table 1). Even comparing within developmental stage, our results indicate that ethanol treated larvae are hypoactive relative to control (non-treated) larvae when activity is assessed based on the episode frequency (Table 4). However, our results also indicate that ethanol treated larvae are hyperactive relative to control (non-treated) larvae when activity is assessed based on swim speed or episode duration (Table 4). Although these measures are relevant attributes of locomotor activity, we find that no single measure fully depicts the activity level and thus different interpretations of locomotor activity can be made. It is also important to recognize that, although the larvae used for this study were reared in a group setting, the data reported here are based exclusively on larvae isolated in individual wells. Thus, group interactions are not present in this experimental paradigm and, therefore, these results cannot be extended to paradigms that examine larval zebrafish locomotor activity in various group settings.

We conclude that high-throughput experiments that measure and assess locomotor activity in larval zebrafish benefit from the reduced variability observed when older larvae (7 dpf) in deep (5.5 mm) wells are used. In addition, since we have shown that a single measure of locomotor activity (e.g., swim speed or total distance traveled) is not sufficient to fully describe activity level, we suggest that a more detailed assessment of the effects on activity level requires the evaluation and integration of multiple properties of locomotor activity.

### Conflict of interest statement

The authors declare that the research was conducted in the absence of any commercial or financial relationships that could be construed as a potential conflict of interest.
